# Plasma-Engineered PDRN: Surface Charge Neutralization and Nanosizing Enhance Uptake and Regeneration Potential

**DOI:** 10.3390/pharmaceutics17091136

**Published:** 2025-08-30

**Authors:** Sun Ju Park, Dong-Hwan Lee, Ki Bok Yoon, AhJin Kim, Chae-Yun Jung, Sung Tae Kim, Sofia Brito, Bum-Ho Bin

**Affiliations:** 1 Department of Biological Sciences, Ajou University, Suwon 16499, Republic of Korea; 2 Department of Nanoscience and Engineering, Inje University, Gimhae 50834, Republic of Korea; 3 Department of Pharmaceutical Engineering, Inje University, Gimhae 50834, Republic of Korea; 4 Department of Pharmacology, College of Medicine, The Catholic University of Korea, Seoul 06591, Republic of Korea; 5 The Anti-Aging Lab, Co. Ltd., Suwon 16499, Republic of Korea

**Keywords:** PDRN, plasma treatment, surface charge, nanosizing, cosmetic ingredient

## Abstract

**Background**: Polydeoxyribonucleotide (PDRN) is increasingly used in dermatology and cosmetic applications owing to its regenerative and anti-aging properties. However, its topical use is limited by its high molecular weight and anionic charge, which restrict skin penetration. **Methods**: In this study, we employed a nitrogen-oxygen plasma treatment to PDRN to overcome these limitations and characterized its physicochemical properties and in vitro efficiency. **Results**: Upon plasma treatment, PDRN’s surface charge was attenuated and its hydrodynamic size decreased, leading to improved uptake and markedly increased cell migration activity. **Conclusions**: These findings suggest that plasma treatment can transform PDRN into a cosmetically viable active ingredient and may provide a general strategy for adapting other high-molecular-weight bioactives for topical delivery.

## 1. Introduction

Polydeoxyribonucleotide (PDRN), a DNA-derived polymer primarily extracted from salmon sperm, has garnered significant attention in the fields of regenerative medicine and cosmetics for its skin-regenerative and anti-aging effects, supported by pro-repair and anti-inflammatory activity [1,2,3]. In dermatology, PDRN has been used for wound healing, scar management, and post-procedural recovery, among other uses, due to its ability to activate the A2A adenosine receptor pathway and stimulate fibroblast proliferation and extracellular matrix (ECM) synthesis [4,5,6,7,8,9,10]. PDRN comprises polynucleotide fragments of approximately 50–1500 kDa, a size range that precludes passive diffusion across the stratum corneum [11]; by contrast, efficient passive skin permeation is generally limited to <500 Da molecules [12]. Moreover, nucleic acids are poorly taken up by cells because of their negative charge and hydrophilicity [13]. Additionally, PDRN’s anionic charge may limit passive diffusion across the stratum corneum and hinder cellular uptake, resulting in poor topical bioavailability [14]. Consequently, unmodified PDRN is suboptimal as a cosmetic active ingredient, and strategies that reduce its size and/or modulate its surface charge are required to enable effective delivery.

Plasma is a partially ionized gas composed of free electrons, charged ions, and chemically reactive species such as oxygen and nitrogen radicals [15,16,17,18,19,20]. Because of these species, plasma can modify surface properties and functionalize biomaterials (e.g., surface charge, hydrophilicity, roughness, and related features) [21,22]. Because of these properties, in recent years, plasma has emerged as a versatile tool in biomedical and nanomedicine applications, including device sterilization, polymer coating, nanoparticle functionalization, and the modification of tissue engineering scaffolds [23,24]. Therefore, plasma has the potential to improve the stability, delivery, and cellular uptake of PDRN by enhancing its surface characteristics and promoting better interactions with biological systems.

In the present work, we hypothesize that nitrogen-oxygen plasma processing may reduce the hydrodynamic size of PDRN and attenuate its anionic surface charge, thereby enhancing cellular uptake and topical performance. Specifically, nanosizing is expected to increase diffusivity, whereas charge attenuation should lessen electrostatic repulsion with cell membranes and barrier components. We test this by characterizing physicochemical changes and assessing cellular permeability, skin permeation, and fibroblast migration. Our findings highlight the potential of plasma-assisted processing as a novel platform to enable the cosmetic application of high-molecular-weight biopolymers such as PDRN.

## 2. Materials and Methods

### 2.1. Materials

Polydeoxyribonucleotide (PDRN, >95% purity) was purchased from Pharma Food Korea (Seoul, Republic of Korea). All other reagents, including phosphate-buffered saline (PBS), Dulbecco’s Modified Eagle Medium (DMEM), fetal bovine serum (FBS), and trypsin-EDTA, were obtained from Gibco (Thermo Fisher Scientific, Waltham, MA, USA) unless otherwise stated. Human dermal fibroblasts (HDFs) were cultured in DMEM supplemented with 10% FBS and 1% penicillin-streptomycin at 37 °C under 5% CO_2_.

### 2.2. Plasma Modification of PDRN

**Figure 1:**
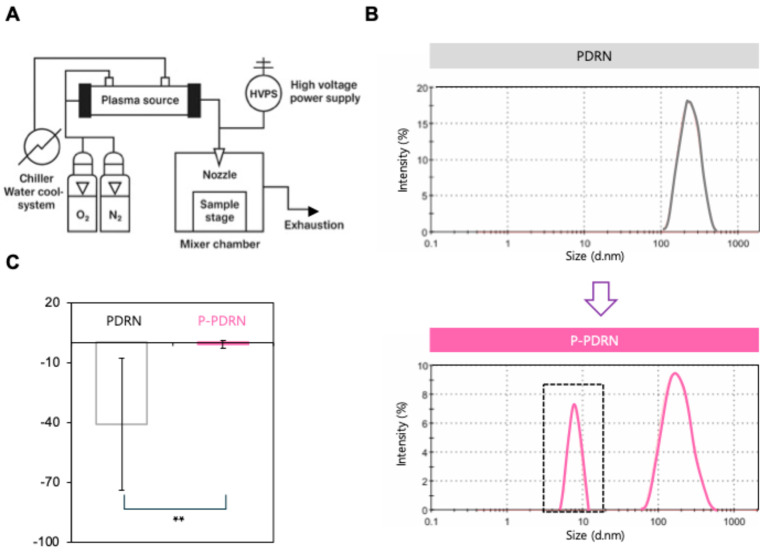
Generation and characterization of P-PDRN. (**A**) Schematic illustration of the plasma device utilized for the treatment of PDRN. (**B**) Hydrodynamic size distributions of PDRN. Native PDRN (black trace) shows a monomodal peak centered at ~300 nm, whereas P-PDRN (pink trace) shows a bimodal profile with an additional population at ~10 nm (highlighted by the black dashed box). (**C**) Zeta potential measurement of PDRN and plasma-treated PDRN (P-PDRN). Untreated PDRN exhibited a strongly negative surface charge (−34 mV), while P-PDRN showed a positive charge (+6.3 mV), confirming successful surface charge inversion (*n* = 10, ** *p* < 0.01).

A custom-built atmospheric-pressure plasma gas injection device (Figure 1A) was used to treat the PDRN solution. The system comprised a plasma source connected to a high-voltage power supply (HVPS) of 500 W, a chiller-based water-cooling unit, and gas supply lines for both O_2_ and N_2_. Plasma was generated with a voltage of 6.5 kV and frequency of 30 kHz, with a N_2_ flow rate of 1 L/min each and an O_2_ flow rate of 0.11 L/min. The solution (1 mg/mL PDRN in distilled water in an Eppendorf tube) was placed in a sealed mixer chamber on a temperature-controlled stage (25 ± 2 °C) and exposed to plasma for 5 min at atmospheric pressure. Immediately after treatment, the plasma-modified PDRN (P-PDRN) was collected and stored at 4 °C until use.

### 2.3. Particle Size

The hydrodynamic size, size distribution, and polydispersity (PDI) of PDRN and P-PDRN were measured using dynamic light scattering (DLS; Zetasizer Nano ZS, Malvern Instruments, Malvern, UK) with disposable folded capillary cells. The dispersant was triple-distilled water (software refractive index = 1.333); the temperature was 25 ± 2 °C. Each sample was analyzed within 30 min of plasma treatment.

### 2.4. Zeta Potential Analysis

Zeta potential was measured by electrophoretic light scattering (ELS) on a nanoPartica SZ-100V2 analyzer (Horiba, Kyoto, Japan) with disposable folded capillary cells. The dwas triple-distilled water (software refractive index = 1.333); the temperature was 25 ± 2 °C. Analyses were conducted in triplicate with seven consecutive runs. Each sample was analyzed within 30 min of plasma treatment.

### 2.5. Fourier-Transform Infrared (FT-IR) Spectroscopy

Both PDRN and plasma-treated PDRN (P-PDRN) were subjected to lyophilization under vacuum at −60 °C using a freeze dryer (TFD5503, iLShin Bio Base Co., Ltd., Dongducheon, Republic of Korea) prior to Fourier transform infrared (FT-IR) spectroscopy. The lyophilized powders were thoroughly ground and homogeneously mixed with spectroscopic-grade potassium bromide (KBr) at a weight ratio of approximately 1:30 (sample:KBr). The mixtures were then compressed into transparent pellets using a hydraulic press. Each FT-IR spectrium was acquired over the wavenumber range of 4000–400 cm^−1^, with a resolution of 4 cm^−1^ and 32 accumulative scans, using a Varian 640-IR spectrometer (Agilent Technologies, Santa Clara, CA, USA). Spectra were collected in transmission mode, and baseline correction was applied prior to data interpretation.

### 2.6. Cellular Uptake Assay

Cells were seeded onto Tomodishes and incubated with either PDRN or P-PDRN (100 μg/mL) pre-labeled with Hoechst 33258 (Sigma-Aldrich, St. Louis, MO, USA) and incubated for 2 h. For labeling, Hoechst 33258 was mixed with PDRN or P-PDRN for 20 min at 37 °C, and unbound dye was removed with a 3.5K MWCO dialysis cassette (Slide-A-Lyzer, Thermo Fisher Scientific Waltham, MA, USA) overnight. Imaging was performed on a Tomocube HT-2H (Tomocube Inc., Daejeon, Republic of Korea); refractive-index tomograms and the corresponding fluorescence channel were processed in TomoStudio software v. 3.3.9 with identical settings across conditions. For quantification, fluorescence-only images were exported against a black background. Fluorescence quantification was performed in ImageJ 1.54g using fixed-size square ROIs placed over intracellular regions to measure the mean gray value, then normalized to the mean of the PDRN control; results are reported as mean ± SD from at least 5 cells per condition.

### 2.7. In Vitro Skin Permeation Analysis

The permeation of PDRN and P-PDRN was assessed using Franz diffusion cells fitted with an artificial skin membrane (Strat-M^®^ Transdermal Diffusion Membrane, Sigma-Aldrich, St. Louis, MI, USA). The receptor compartment was filled with deionized water and maintained at 32 ± 0.5 °C under constant stirring to mimic skin surface temperature and ensure uniform mixing. The donor compartment was loaded with an equivalent concentration of PDRN or P-PDRN in distilled water, and the membrane surface was occluded. At predetermined intervals, 2 µL samples were collected from the receptor compartment, and the nucleic acid content was quantified using a Nano-300 UV-Vis Spectrophotometer (Allsheng, Hangzhou, China). Cumulative permeation (ng/µL) was plotted against time, and linear regression was applied to the steady-state region (60–180 min) to calculate permeation flux (ng/µL·h^−1^).

### 2.8. Cell Migration Assay

HDFs were seeded into 6-well plates and cultured until reaching a confluence of around 90%. A uniform scratch was introduced using a sterile pipette tip, and the wells were washed with PBS to remove detached cells. Cells were then incubated in serum-free medium containing either PDRN or P-PDRN (100 μg/mL). Phase-contrast images were acquired at 0 and 24 h using an inverted microscope (CKS53, Olympus, Tokyo, Japan). The cell-free gap area (A) was measured in ImageJ 1.54g; for each well, the 0 h area was set to 1, and percent closure at 24 h was calculated as Normalized Area Coverage = 1 − A24_norm.

### 2.9. Clinical Evaluation of Cosmetic Formulation Containing P-PDRN

A human trial was commissioned to an independent testing organization to evaluate the cosmetic effects of a cream containing P-PDRN. The study protocol was approved by the Institutional Review Board (IRB Approval No. HM-IRB-P25-0477, Registration date 31 of July 2025). Participants consisted of 21 healthy Korean women with a mean age of 57.81 ± 8.57 years who provided written informed consent after receiving a full explanation of the study’s purpose and procedures. Inclusion criteria required no acute or chronic systemic disease, no skin disorders, and availability for follow-up during the study period. Exclusion criteria included pregnancy, breastfeeding, psychiatric illness, infectious skin diseases, recent steroid use (>1 month), participation in a similar trial within the past 6 months, hypersensitive skin, visible skin abnormalities at the test site, recent use of similar cosmetic/medicinal products (within 3 months), recent dermatological procedures at the test site (within 6 months), employment by the sponsor, or other conditions deemed unsuitable by the investigator. Participants applied the P-PDRN cream to the face or forearm twice daily (morning and evening) for two weeks. Measurements were performed at baseline, after a single application, and after two weeks of use under controlled conditions (25 ± 2 °C, 50 ± 5% relative humidity). Skin surface texture was measured using Antera 3D (Miravex Ltd., Dublin, Ireland), which captures seven-wavelength multispectral images and reconstructs high-resolution 3D skin maps to analyze parameters including the arithmetic mean roughness (Ra, μm) of the cheek area. Skin gloss was assessed using Mark-Vu (PSI Plus Co., Suwon, Republic of Korea) in gloss-light mode, and quantified using the SkinGlossMeter (Delfin Technologies Ltd., Kuopio, Finland), which measures reflected light from a 635 nm diode laser (50 μm spot size). Results were expressed in arbitrary units (A.U.), with higher values indicating increased gloss. Analyses (SPSS v26) used repeated-measures ANOVA with Bonferroni correction, or Friedman/Wilcoxon if Shapiro–Wilk normality was violated; data are mean ± SD or n (%); α = 0.05 (two-sided).

### 2.10. Statistical Analysis

Data are presented as mean ± standard deviation (SD). Except for the clinical evaluation (Section 2.9), the statistical significance for all experiments, unless stated, was determined using a two-tailed Student’s *t*-test. Significance was defined as * *p* < 0.05, ** *p* < 0.01, *** *p* < 0.001.

## 3. Results

### 3.1. Plasma-Induced Nanosizing and Surface Charge Modulation of PDRN

To investigate the physicochemical changes of PDRN induced by nitrogen-oxygen plasma treatment, we exposed the PDRN solution to atmospheric-pressure plasma generated from a biocompatible gas mixture of nitrogen (N_2_) and oxygen (O_2_) ejected by a custom-built plasma gas injection device (Figure 1A) [25]. We proposed that the reactive ions and radicals produced by the nitrogen-oxygen plasma would interact with the PDRN molecules, forming a plasma-modified PDRN (P-PDRN) while preserving the structural integrity of the biopolymer due to the mild and biocompatible nature of the treatment. Such surface modification is anticipated to occur without significant thermal degradation or structural damage, owing to the mild and biocompatible nature of the nitrogen-oxygen plasma. The particle size distribution of untreated and plasma-treated PDRN was evaluated using DLS analysis. To investigate whether plasma treatment induces structural alterations in PDRN, FT-IR spectroscopy was conducted (Figure S1A). Compared with untreated PDRN, P-PDRN displayed distinct spectral changes, including the emergence of a peak at 1696.7 cm^−1^ (C=O stretching, indicative of base oxidation), enhanced intensity at 1384.1 cm^−1^ (associated with base ring exposure), and modifications in the phosphate region, such as the appearance of a peak at 529.5 cm^−1^ (free PO_4_^3−^ groups) and a shift at 1062.2 cm^−1^ (phosphate bonding changes). These results suggest that plasma treatment introduces surface-level chemical modifications, presumably involving some degree of chain scission and altered charge distribution, while largely preserving the DNA backbone and thereby maintaining functional activity. The untreated PDRN exhibited a monodisperse peak centered around ~300 nm, indicating a relatively large molecular size typical of high-molecular-weight DNA polymers (Figure 1B; upper graph). However, after plasma exposure, the DLS profile revealed the appearance of a distinct new peak at ~10 nm (Figure 1B; bottom graph, dashed box), suggesting a significant population of smaller molecular fragments by the nanosizing of PDRN. To evaluate the effect of plasma treatment on the surface charge of PDRN, the zeta potential of both untreated and plasma-treated samples was measured. Untreated PDRN exhibited a strongly negative surface charge, with an average zeta potential of approximately −40.98 ± 33 mV, consistent with the inherent phosphate backbone of the DNA molecule (Figure 1C). In contrast, after nitrogen-oxygen plasma exposure, the zeta potential shifted significantly toward the positive direction, reaching −1.29 ± 1.99 mV. To understand the stability of plasma treatment, we analyzed zeta potential, particle size, and polydispersity index (PDI) over time. Zeta potential measurements showed no marked fluctuations over the 24 h testing period (Figure S1B), suggesting that surface charge neutralization induced by plasma treatment is preserved over time. The results also indicated that the nanosized population generated by plasma treatment was maintained up to 24 h without significant aggregation or broadening of the size distribution (Figure S1C). Lastly, PDI values showed moderate monodispersity at *t*_0_ (0.07 ± 0.01) [26], with no significant changes over time, indicating a uniform and stable particle population (Figure S1D). Collectively, these results demonstrate that nitrogen–oxygen plasma treatment induces surface-level chemical modifications, charge neutralization, and nanosizing of PDRN while preserving its structural integrity and short-term physicochemical stability, thereby generating a functionally active plasma-engineered formulation.

### 3.2. Enhanced Uptake and Permeation of Plasma-Treated PDRN

**Figure 2:**
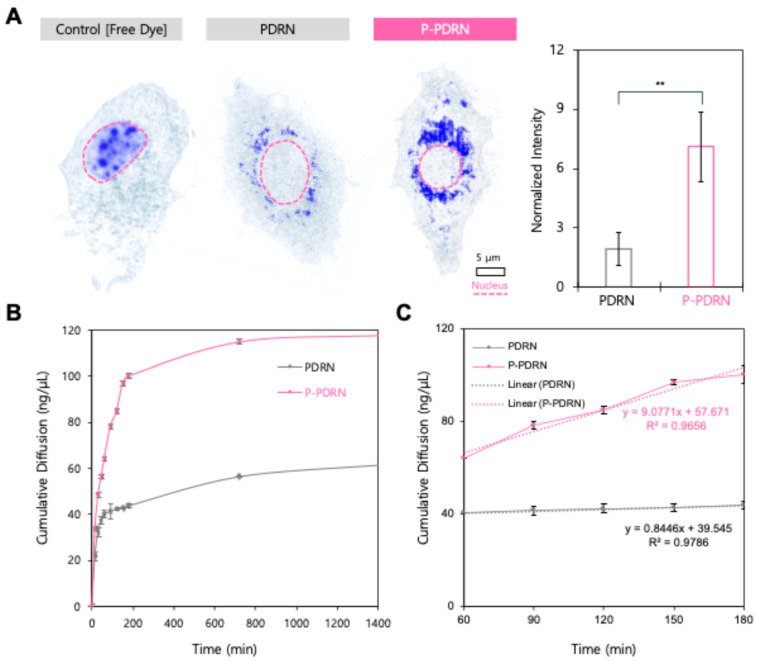
P-PDRN shows enhanced uptake and permeation. (**A**) Holotomography images of HDFs treated with control free dye, PDRN, or plasma-treated PDRN (P-PDRN). Blue signal indicates intracellular localization of PDRN. Scale bar = 5 μm. The graph on the right shows a quantification of intracellular PDRN intensity (** *p* < 0.01). (**B**) Cumulative permeation profiles of PDRN and P-PDRN across a Strat-M^®^ artificial skin membrane in Franz diffusion cells. Data represent mean ± SD (*n* = 3). P-PDRN demonstrated consistently higher permeation than PDRN over 24 h. (**C**) Subset of panel B showing the steady-state region (60–180 min) used for flux calculations, with linear regression equations and R^2^ values indicating ~10.7-fold greater flux for P-PDRN.

To evaluate the effect of plasma treatment on the cellular uptake of PDRN, we visualized the intracellular distribution of PDRN and P-PDRN using holotomography imaging (Figure 2A). In PDRN-treated cells, a few punctate signals were detected within the cytoplasm, indicating limited uptake. In contrast, cells treated with P-PDRN exhibited strong and widespread intracellular signal intensity, particularly concentrated in the perinuclear region, suggesting enhanced cellular internalization of plasma-modified PDRN. Quantitative analysis of intracellular signal intensity (Figure 2B) confirmed these observations. The mean intracellular fluorescence of the P-PDRN group was significantly higher than that of the untreated PDRN group (*p* < 0.01), demonstrating that nitrogen-oxygen plasma processing effectively improves cellular uptake efficiency. These findings support the hypothesis that plasma-induced size reduction and surface charge inversion promote cellular permeability, likely by overcoming electrostatic repulsion at the cell membrane and facilitating endocytic entry.

Next, we analyzed the cumulative permeation profiles of PDRN and P-PDRN across an artificial skin model (Figure 2B). P-PDRN exhibited markedly greater permeation than untreated PDRN at all time points. Within the first 180 min, P-PDRN reached 100.22 ± 2.52 ng/µL, corresponding to a ~2.29-fold higher cumulative permeation than PDRN (43.76 ± 1.04 ng/µL). The P-PDRN profile showed a steep initial rise followed by a gradual plateau, consistent with rapid diffusion and subsequent saturation of the membrane. In contrast, PDRN permeated more slowly and reached a near-steady state at approximately 150 min. This enhanced permeation is likely mainly attributable to the smaller particle size of P-PDRN generated by plasma treatment, which increases the surface area-to-volume ratio and facilitates faster barrier penetration. Linear regression analysis of the steady-state region (60–180 min; Figure 2C) revealed a flux of 544.6 ng/µL·h^−1^ for P-PDRN (slope = 9.0771 ng/µL·min^−1^; R^2^ = 0.9656), compared with 50.7 ng/µL·h^−1^ for PDRN (slope = 0.8446 ng/µL·min^−1^; R^2^ = 0.9786), indicating a ~10.7-fold higher permeation rate for the plasma-treated formulation.

Together, these results demonstrate that plasma treatment markedly enhances both cellular uptake and transdermal permeation of PDRN, highlighting its potential as an improved therapeutic formulation for skin regeneration.

### 3.3. Plasma-Treated PDRN Accelerates Fibroblast Migration and Shows Potential as a Skin Regeneration Cosmetic

**Figure 3:**
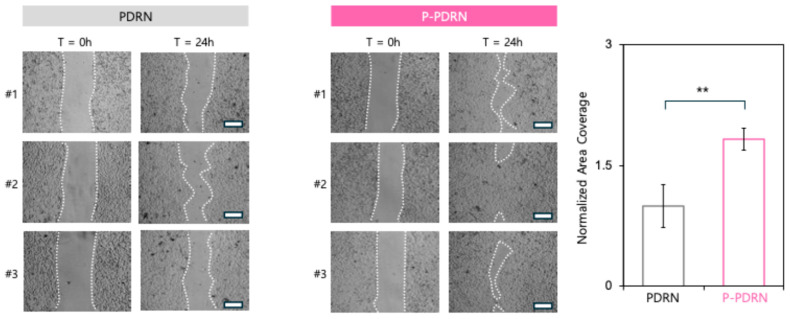
P-PDRN enhances fibroblast migration. Scratch assay images of HDFs treated with PDRN (**left**) or P-PDRN (**right**) at 0 and 24 h. White dotted lines represent the wound edges. Faster wound closure is observed in the P-PDRN group, indicating enhanced cell migration and regenerative activity. Scale bar: 100 μm. The graph on the right shows a quantification of the normalized area coverage (** *p* < 0.01).

To assess the biological activity of plasma-treated PDRN (P-PDRN), a cell migration assay was conducted using human dermal fibroblasts. Representative images of cell migration to the scratched area at 0 and 24 h are shown in Figure 3. Cells treated with PDRN exhibited gradual migration toward the wound site over 24 h, with only partial closure observed at the final time point (Figure 3). In contrast, cells treated with P-PDRN demonstrated significantly enhanced wound closure, with accelerated edge advancement and narrowing of the scratch gap, indicating increased cell motility and migration. Quantification of wound closure further solidified the enhanced properties of P-PDRN. These results suggest that nitrogen-oxygen plasma treatment of PDRN not only improves physicochemical properties such as cellular uptake but also enhances its biological efficacy in promoting fibroblast migration, a key process in skin regeneration and wound repair.

To further assess the cosmetic potential of P-PDRN, a human clinical study using a cosmetic cream formulation containing P-PDRN was conducted (Figure S2A). In this trial, the product was applied to the facial skin or forearm of volunteers, and parameters related to skin texture and radiance were evaluated before application, after a single application, and after two weeks of twice-daily application. Skin texture was analyzed using an Antera 3D imaging system texture (Figure S2B). The quantitative analysis of the arithmetic mean roughness (Ra, μm) revealed a progressive reduction in skin roughness over the treatment period, with statistically significant improvement observed after two weeks of use. Skin gloss and radiance were assessed using a Mark-Vu facial imaging system in gloss mode (Figure S2C). The quantification after product application confirmed an increase in skin gloss, with significant enhancement maintained after two weeks. These findings indicate that P-PDRN-containing cream can improve skin texture and radiance within a short application period, supporting its value as a functional cosmetic ingredient.

## 4. Discussion

In this study, we demonstrated that nitrogen-oxygen plasma treatment enhances the physicochemical and biological properties of PDRN, a high-molecular-weight DNA polymer widely used in cosmetics and regenerative medicine. Plasma treatment reduced the apparent hydrodynamic size of PDRN, evidenced by the emergence of a distinct ~10-nm population. This substantial size decrease is most consistent with limited polymer chain scission by plasma-generated RONS together with concurrent surface functionalization that attenuates charge, providing electrostatic stabilization and suppressing re-aggregation. Such nanosizing is advantageous for cosmetic or transdermal applications, where particle dimensions strongly influence diffusion across the stratum corneum [27,28,29]. While these data support surface-level modification rather than wholesale degradation, definitive chemical assignments are left for targeted spectroscopic analyses in future work. Zeta potential measurements indicate attenuation of surface charge, consistent with surface functionalization by plasma-generated RONS that reduce PDRN’s anionic properties. This charge reduction is expected to decrease electrostatic repulsion with the cell membrane and promote cellular interaction and uptake [27,30,31,32]. FT-IR features indicate plasma-induced surface modifications that plausibly reduce the net negative charge of PDRN. While informative of carbonyl formation (1696.7 cm^−1^) and phosphate-region changes (~1062 and 529 cm^−1^), the present results do not distinguish true covalent neutralization from other surface processes. A definitive assignment will require orthogonal methods such as high-resolution XPS (P 2p/N 1s), solution-state ^31P NMR, and LC-MS/MS of enzymatic digests. Targeted analyses will help identify the modifications more precisely. Consistent with nanosizing and zeta potential attenuation, holotomography imaging showed the markedly improved cellular uptake of P-PDRN. This aligns with prior evidence reporting that particle size and surface potential govern endocytic entry efficiency and intracellular trafficking [33,34]. Permeation testing showed consistently higher cumulative permeation and a greater steady-state flux for P-PDRN than PDRN. This improvement aligns with its uniform ~10-nm size, low PDI, and attenuated surface charge, which together increase diffusivity and reduce electrostatic hindrance during transport. Functionally, this enhanced delivery translated into a biological effect as observed by fibroblastic migration assays, in which P-PDRN accelerated gap closure relative to PDRN. Together, these findings suggest that nitrogen-oxygen plasma-treated PDRN retains or even amplifies its bioactivity post-modification and may serve as a superior functional ingredient in cosmetic and therapeutic contexts.

In this study, nitrogen-oxygen plasma was selected over alternative modification techniques such as chemical functionalization or ultrasonication due to its combined safety, efficiency, and versatility. Plasma treatment is inherently solvent-free, reagent-free, and non-contact, avoiding the introduction of potentially cytotoxic or non-physiological chemicals that may require extensive post-treatment purification. The use of nitrogen and oxygen, both naturally occurring in the human body and biocompatible as atmospheric gases, minimizes the risk of residual contaminants that could compromise safety or bioactivity. Moreover, nitrogen-oxygen plasma uniquely enables simultaneous surface activation and nanoscale fragmentation in a single step under mild temperature conditions. In contrast, chemical functionalization typically requires multiple reaction and purification stages, while ultrasonication is limited to size reduction and may induce uncontrolled degradation with prolonged exposure. This dual modification capability, combined with its rapid, scalable, and environmentally friendly nature, makes nitrogen–oxygen plasma particularly suitable for industrial translation without generating hazardous waste.

## 5. Conclusions

In conclusion, we show that plasma treatment offers potential for the modification of cosmetic actives, enabling the controlled tuning of size and surface charge under mild conditions. In particular, nitrogen-oxygen plasma provides a simple, biocompatible, and efficient route to modify PDRN, a widely used cosmetic polymer. The reagent-free, scalable nature of this approach makes it attractive for product development. Future work will delineate the precise chemical changes and assess durability across additional formulations.

## Data Availability

The raw data supporting the conclusions of this article will be made available by the authors on request.

## Supplementary Materials

The following supporting information can be downloaded at: https://www.mdpi.com/article/10.3390/pharmaceutics17091136/s1, Figure S1: Physicochemical characterization and stability of P-PDRN; Figure S2: Clinical evaluation of a cosmetic cream containing P-PDRN.

## Abbreviations

The following abbreviations are used in this manuscript:

PDRN	Polydeoxyribonucleotide
HDFs	Human Dermal Fibroblasts
P-PDRN	Plasma-modified PDRN
DLS	Dynamic Light Scattering
RONS	Reactive oxygen and nitrogen species
FT-IR	Fourier-transform infrared
