# Supplementary material for: Plasma-Engineered PDRN: Surface Charge Neutralization and Nanosizing Enhance Uptake and Regeneration Potential
Source: Pharmaceutics. 2025 Aug 30;17(9):1136. doi: 10.3390/pharmaceutics17091136 (PMC12473307; doi:10.3390/pharmaceutics17091136)
Supplement: Supplementary file 1 [file pharmaceutics-17-01136-s001.zip › pharmaceutics-3803582-supplementary.pdf]

pharmaceutics

MDPI

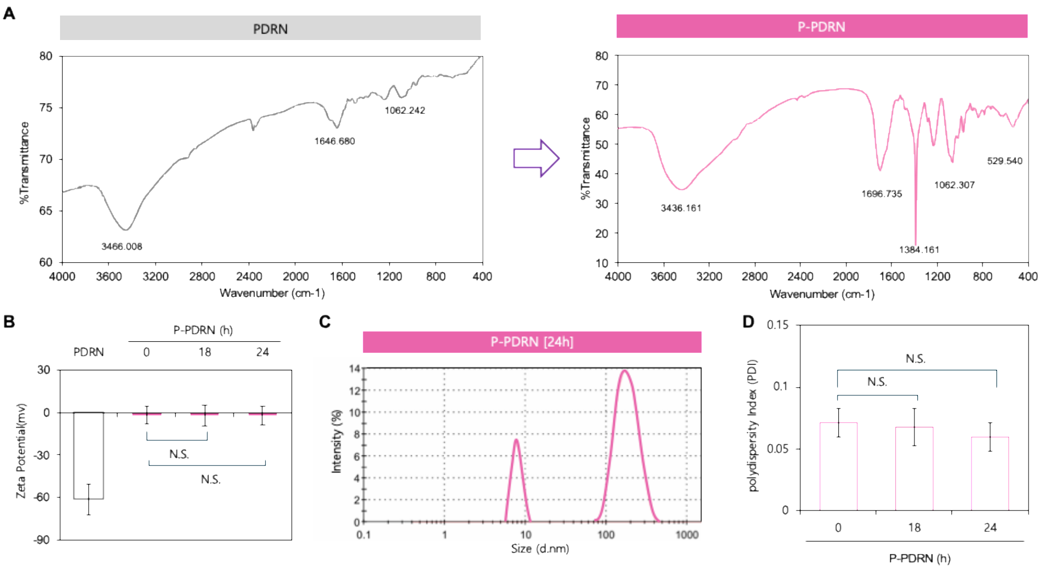

Figure S1. Physicochemical characterization and stability of P-PDRN. (A) FT-IR spectra of untreated PDRN and P-PDRN showing plasma-induced changes. (B) Zeta potential of P-PDRN over time. (C) Size distribution profile of P-PDRN measured by dynamic light scattering after 24 h. (D) Polydispersity index (PDI) of P-PDRN measured at different time points.

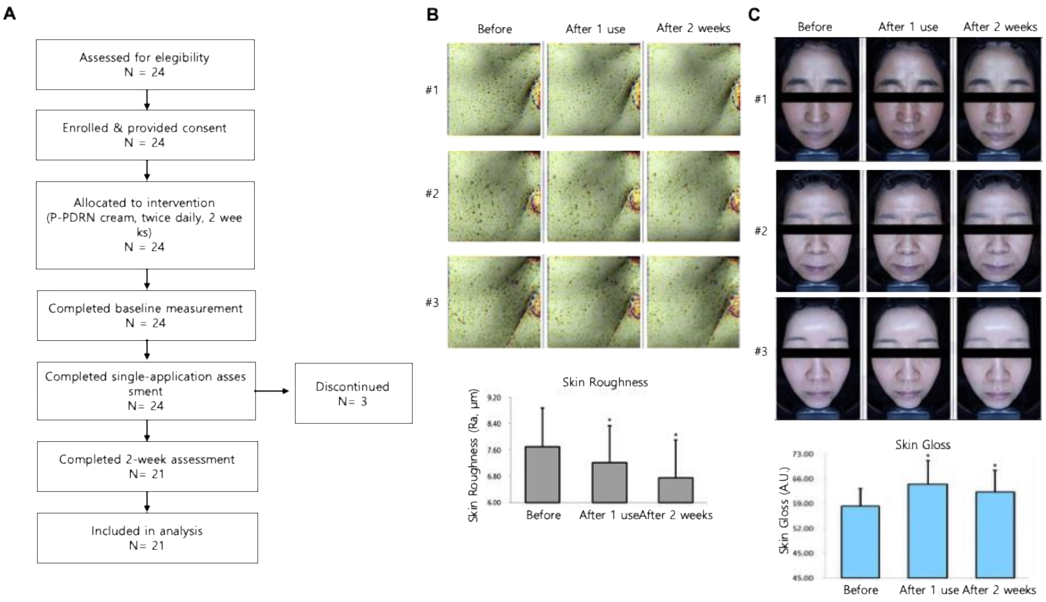

Figure S2. Clinical evaluation of a cosmetic cream containing P-PDRN. (A) Flow chart diagram of the clinical experiment. (B) Representative Antera 3D multispectral texture maps acquired at baseline, after a single application, and after 2 weeks of twice-daily use (cheek/forearm test sites). The graph below represents the arithmetic mean roughness (Ra, μm) quantified from the same regions. (C) Representative Mark-Vu gloss-mode facial images at the same time points. The graph on the right represents skin gloss quantified with a SkinGlossMeter and expressed in arbitrary units (A.U.; higher values indicate greater radiance). Data are mean ± SD (n = 21), (*$p < 0.05$).
